# Supplementary material for: Liver X receptor unlinks intestinal regeneration and tumorigenesis
Source: Nature. 2024 Nov 20;637(8048):1198–206. doi: 10.1038/s41586-024-08247-6 (PMC11779645; doi:10.1038/s41586-024-08247-6)
Supplement: Supplementary file 1 — Supplementary Figs. 1–3, including gating strategies (Supplementary Figs. 1 and 2), uncropped scans of immunoblots (Supplementary Fig. 3) and information about Supplementary Videos 1–5. [file 41586_2024_8247_MOESM1_ESM.pdf]

nature portfolio

https://doi.org/10.1038/s41586-024-08247-6

Supplementary information

Liver X receptor unlinks intestinal regeneration and tumorigenesis

In the format provided by the authors and unedited

This file contains a Supplementary Figures 1-3, which includes gating strategies (Supplementary Figures 1 and 2), uncropped scans of immunoblots (Supplementary Figure 3) and information about Supplementary videos (Supplementary Videos 1-5).

Supplementary Fig. 1

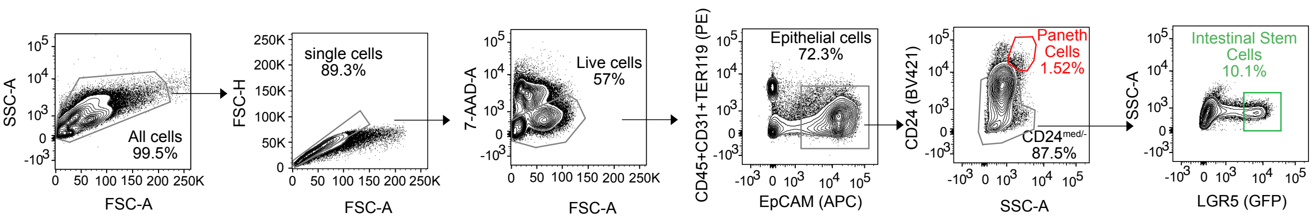

Supplementary Figure 1: FACS gating strategy

Representative FACS gating strattegy for sorting Lgr5-EGFP+ intestinal stem cells and Paneth cells out of small intestial crypt isolates. All data is presented as contour plots with 5% outliers. Fluorophores used are represented in the axis labels. Related to Extended Data Fig. 4b.

Supplementary Fig. 2

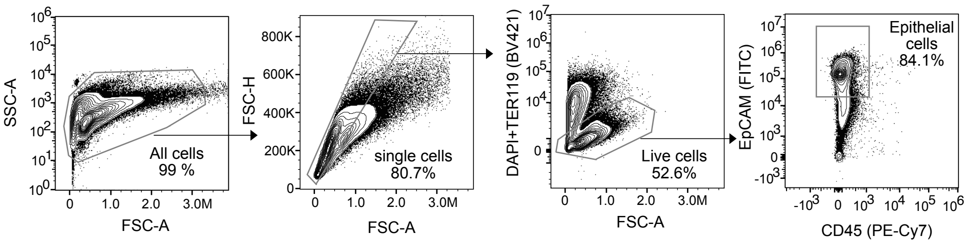

Supplementary Figure 2: FACS gating strategy.

Representative FACS gating strattegy for sorting EpCAM+ intestinal epithelial cells out of small intestial organoid cultures. All data is presented as contour plots with 5% outliers. Fluorophores used are represented in the axis labels. Related to Fig. 2g-i and Extended Data Fig. 5.

Supplementary Fig. 3

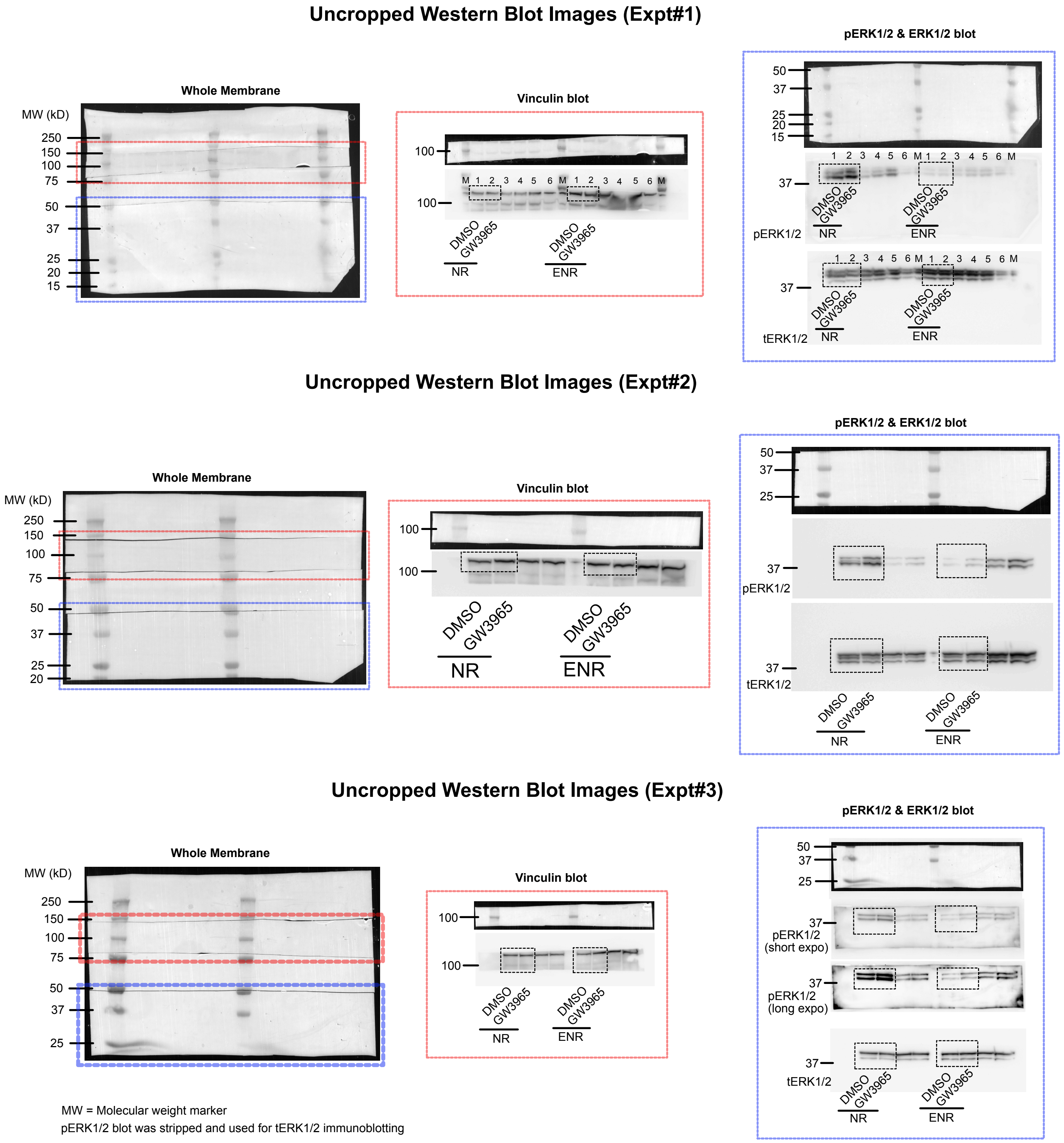

Supplementary Figure 3: Uncropped scans of immunoblots.

Uncropped immunoblotes related to Extended Data Fig. 4h. pERK1/2 blots were stripped and used for total ERK1/2 (tERK1/2) immunoblotting. Of the three independent experiments, black dotted lines from Expt#2 is used in the paper Extended Data Fig. 4h. MW, Molecular weight marker. The loading control marker vinculin was run in the same gel, which was cut out (due to molecular weight difference with other proteins of interest). For ERK1/2, the membrane used for pERK1/2 was stripped off and were reblotted tERK1/2.

Supplementary Video 1: Time-lapse video of single cell replating of mouse small intestinal (SI) organoids grown in ENR (EGF, Noggin, R-spondin) medium and treated with DMSO.
SI crypts were isolated from WT mice and cultured in ENR with DMSO for 5-7 days (primary organoids). Organoids were then digested to single cell suspension and 10,000 live cells were re-seeded for secondary organoid culture. Secondary organoids were cultured in ENR DMSO and were imaged longitudinally every 6 hour till day 6, using Incucyte live imaging system. Related to Fig. Extended Data Fig. 4g and Fig. 2d-f.

Supplementary Video 2: Time-lapse video of single cell replating of mouse small intestinal (SI) organoids grown in NR (Noggin, R-spondin) medium and treated with DMSO.
SI crypts were isolated from WT mice and cultured in NR with DMSO for 5-7 days (primary organoids). Organoids were then digested to single cell suspension and 10,000 live cells were re-seeded for secondary organoid culture. Secondary organoids were cultured in NR DMSO and were imaged longitudinally every 6 hour till day 6, using Incucyte live imaging system. Related to Fig. Extended Data Fig. 4g and Fig. 2d-f.

Supplementary Video 3: Time-lapse video of single cell replating of mouse small intestinal (SI) organoids grown in NR (Noggin, R-spondin) medium and treated with GW3965.
SI crypts were isolated from WT mice and cultured in NR with GW3965 for 5-7 days (primary organoids). Organoids were then digested to single cell suspension and 10,000 live cells were re-seeded for secondary organoid culture. Secondary organoids were cultured in NR GW3965 and were imaged longitudinally every 6 hour till day 6, using Incucyte live imaging system. Related to Fig. Extended Data Fig. 4g and Fig. 2d-f.

Supplementary Video 4: Time-lapse video of single cell replating of mouse colonic organoids treated with DMSO.
WT colon primary organoids were cultured for 5-6 days with DMSO. Organoids were then split and 10,000 live colonocytes were re-seeded for secondary organoids, treated with DMSO and were imaged longitudinally every 4 hour for 6 days with Incucyte live imaging system. Related to Fig. Extended Data Fig. 8d-g.

Supplementary Video 5: Time-lapse video of single cell replating of mouse colonic organoids treated with GW3965.
WT colon primary organoids were cultured for 5-6 days with GW3965. Organoids were then split and 10,000 live colonocytes were re-seeded for secondary organoids, treated with GW3965 and were imaged longitudinally every 4 hour for 6 days with Incucyte live imaging system. Related to Fig. Extended Data Fig. 8d-g.
