# Supplementary material for: Liver X receptor unlinks intestinal regeneration and tumorigenesis
Source: Nature. 2024 Nov 20;637(8048):1198–206. doi: 10.1038/s41586-024-08247-6 (PMC11779645; doi:10.1038/s41586-024-08247-6)
Supplement: Supplementary file 2 — Reporting Summary [file 41586_2024_8247_MOESM2_ESM.pdf]

nature portfolio

Corresponding author(s): Eduardo J. Villablanca

Last updated by author(s): 25/07/2024

Reporting Summary

Nature Portfolio wishes to improve the reproducibility of the work that we publish. This form provides structure for consistency and transparency in reporting. For further information on Nature Portfolio policies, see our Editorial Policies and the Editorial Policy Checklist.

Statistics

For all statistical analyses, confirm that the following items are present in the figure legend, table legend, main text, or Methods section.

n/a	Confirmed	
☐	☒	The exact sample size (*n*) for each experimental group/condition, given as a discrete number and unit of measurement
☐	☒	A statement on whether measurements were taken from distinct samples or whether the same sample was measured repeatedly
☐	☒	The statistical test(s) used AND whether they are one- or two-sided<br>*Only common tests should be described solely by name; describe more complex techniques in the Methods section.*
☒	☐	A description of all covariates tested
☐	☒	A description of any assumptions or corrections, such as tests of normality and adjustment for multiple comparisons
☐	☒	A full description of the statistical parameters including central tendency (e.g. means) or other basic estimates (e.g. regression coefficient) AND variation (e.g. standard deviation) or associated estimates of uncertainty (e.g. confidence intervals)
☐	☒	For null hypothesis testing, the test statistic (e.g. *F*, *t*, *r*) with confidence intervals, effect sizes, degrees of freedom and *P* value noted<br>*Give P values as exact values whenever suitable.*
☒	☐	For Bayesian analysis, information on the choice of priors and Markov chain Monte Carlo settings
☒	☐	For hierarchical and complex designs, identification of the appropriate level for tests and full reporting of outcomes
☒	☐	Estimates of effect sizes (e.g. Cohen's *d*, Pearson's *r*), indicating how they were calculated

Our web collection on statistics for biologists contains articles on many of the points above.

Software and code

Policy information about availability of computer code

Data collection	Flow cytometry: FACSDiva (BD), LSR Fortessa(BD)<br>Floresence activated cell sorting: SH800S(SONY), FACS ARIA II (BD)<br>Confocal microscope: LSM880 (Zeiss)<br>Cell culture imaging: Incucyte S3 Live-Cell Analysis Instrument (Sartorius) (v2023A)<br>qRT-PCR: CFX 384 Realtime C1000 Touch (BioRad)<br>Next Generation Sequencing: 10X chromium Controller, NovaSeq S1 flow cell (Illumina)
Data analysis	GraphPad Prism 9, version 9.5.0<br>Microsoft Excel 16.51<br>FlowJo v10<br>QuPath 0.2.3<br>Fiji (ImageJ2, version:2.14.0/1.54f)<br>R (versions stated in the code)<br>Visiopharm (version 23.01)<br>NDP.View2<br>Incucyte® Software (v2023A)<br>Incucyte® Organoid Analysis Software Module (Cat. No. 9600-0034)<br><br>For further package details please see in methods section and source code repository.

For manuscripts utilizing custom algorithms or software that are central to the research but not yet described in published literature, software must be made available to editors and reviewers. We strongly encourage code deposition in a community repository (e.g. GitHub). See the Nature Portfolio guidelines for submitting code & software for further information.

Data

Policy information about availability of data

All manuscripts must include a data availability statement. This statement should provide the following information, where applicable:

- Accession codes, unique identifiers, or web links for publicly available datasets
- A description of any restrictions on data availability
- For clinical datasets or third party data, please ensure that the statement adheres to our policy

RNAseq data generated in this study has been deposited in the NCBI Gene Expression Omnibus (GEO) repository with the following accession codes. Bulk RNAseq in AOM-DSS CRC experiment (GSE180078), scRNA-seq in organoids (GSE180079), spatial transcriptomics in AOM-DSS CRC experiment (GSE227598), spatial transcriptomics in irradiation induced injury-repair in small intestine (GSE227742) and scRNA-seq of small intestinal cells following LXR activation (GSE227726). All these datasets are now publicly available. All other publicly available datasets used herein are available on GEO with accession codes: GSE14879449, GSE1177834, GSE1310325, GSE3958247. Source data are provided with this paper.

All the custom codes generated in this study have been deposited in a GitHub public repository (https://github.com/ejvillablancaLab/LXR_in_regeneration_and_tumorigenesis), which can be accessed via a DOI at Zenodo: https://doi.org/10.5281/zenodo.13133717.

For further details please see methods section.

Field-specific reporting

Please select the one below that is the best fit for your research. If you are not sure, read the appropriate sections before making your selection.

☒ Life sciences ☐ Behavioural & social sciences ☐ Ecological, evolutionary & environmental sciences

For a reference copy of the document with all sections, see nature.com/documents/nr-reporting-summary-flat.pdf

Life sciences study design

All studies must disclose on these points even when the disclosure is negative.

Sample size	No statistical methods were used to predetermine sample size. To determine the sample size, we conducted pilot experiments to estimate an appropriate number that would ensure significant results in statistical tests. The exact sample sizes used in this study are specified in each figure legend.
Data exclusions	One data point in the control (Cyp27a1+/+ or +/-) group in Fig. 3h, and one data point in the Standard diet group in Extended Data Fig. 10b were excluded which were calculated using the GraphPad outlier calculator for significant outlier in the group by ROUT method. No other data or animals were excluded from analysis, except for clear technical failure.
Replication	10X scRNAseq, spatial transcriptomics in irradiation and CRC, and CRC bulk RNAseq experiments were performed once due to the high costs and intensive resource demands. These techniques capture significant sample heterogeneity, providing insights that can partially substitute for biological replicates. However, for scRNAseq and bulk RNASeq, we used a pool of 2-3 biological replicates which were then treated as single sample for sequencing. For spatial transcriptomics, the findings were further validated in multiple biological replicates using alternative methods.<br><br>All other experiments were repeated as individual experiments as stated in each figure legend, and reproducibility was confirmed.
Randomization	Mice were randomly assigned to experimental groups and received the designated treatments as outlined in the experimental protocol.
Blinding	All the organoids crypt domain quantification was done blindly, meaning the investigator quantifying the organoids was unaware of the genotype and the treatment conditions. Likewise, histology quantification (BrdU, cleaved Cas3, CYP27A1, AREG staining, scoring of DSS colitis and AOM-DSS tumor grading) were done with code-labeled samples.<br><br>For all other experiments, the researchers were not blinded to the treatment or genotypes of the mice to avoid handling errors due to the involvement of multiple scientists. However, the experimental design and use of appropriate controls ensured the accuracy and reproducibility of all measurements and analyses.

Reporting for specific materials, systems and methods

We require information from authors about some types of materials, experimental systems and methods used in many studies. Here, indicate whether each material, system or method listed is relevant to your study. If you are not sure if a list item applies to your research, read the appropriate section before selecting a response.

Materials & experimental systems

n/a	Involved in the study
☐	☒ Antibodies
☒	☐ Eukaryotic cell lines
☒	☐ Palaeontology and archaeology
☐	☒ Animals and other organisms
☐	☒ Human research participants
☒	☐ Clinical data
☒	☐ Dual use research of concern

Methods

n/a	Involved in the study
☒	☐ ChIP-seq
☐	☒ Flow cytometry
☒	☐ MRI-based neuroimaging

Antibodies

Antibodies used

The following antibodies (supplier, clone, catalogue number, dilution) were used for staining and IHC:

CD19-FITC(BioLegend, clone 6D5, Cat#115506, 1:200)
CD45.2-BV650(BioLegend, clone 104, Cat#109836, 1:200)
CD90.2-APC-Cy7(BioLegend, clone 30-H12, Cat#105328, 1:200)
CD64-BV421(BioLegend, clone X54-5/7.1, Cat# 139309, 1:200)
CD11b-BV786(Invitrogen, M1/70, Catalog # 417-0112-82, 1:200)
CD11c-PE-Cy7(BioLegend, clone N418, Cat# 117317, 1:200)
MHC-II-V500(BD-Biosciences, clone M5/114.15.2 , Cat# 562366, 1:200)
Ly6G-PE (BD-Biosciences, clone 1A8, Cat# 551461, 1:200)
Ly6C-PcP5.5(Invitrogen, clone HK1.4, Cat# 45-5932-82, 1:200)
CD3-AF700(BD-Biosciences, clone 500A2, Cat# 557984, 1:200)
CD103-APC(Invitrogen, clone 2E7, Cat# 17-1031-80, 1:200)
CD31-PE, Biolegend, clone: Mec13.3, cat# 102507, 1:500
CD45.2-PE, eBioscience, clone: 30-F11, cat# 12-0451-82, 1:500
Ter-119-PE, Biolegend, clone: Ter119, cat# 116207, 1:500
CD24-PacificBlue, Biolegend, clone: M1/69, cat# 101819, 1:500
EpCAM-APC, eBioscience, clone G8.8, cat# 17-5791-82, 1:500
EpCAM-FITC, Biolegend, clone: G8.8, cat# 118207, 1:200
CD45.2-PeCy7, Biolegend, clone: 104, cat#109829, 1:200
Ter119-Pacific Blue, Biolegend , clone: Ter119, cat# 116231, 1:200
Rat anti-BrdU, Abcam, clone: BU1/75 ICR1, cat# ab6326, 1:200-300
Rabbit anti-cleaved caspase3, Cell Signaling, clone: 5A1E, Cat# 9664S, 1:200
Rabbit anti-CYP27A1, Abcam, clone: EPR7529, Cat# ab126785, 1:200
Mouse anti-AREG, Santacruz Biotechnology, clone: G4, Cat# sc-74501, 1:500
Mouse anti-Vimentin, Abcam, clone: RV202, cat# ab8978, 1:1000
Purified anti-mouse/human CD45R/B220 antibody, Biolegend, Clone: RA3-6B2, cat# 103201, 1:200
Rabbit monoclonal anti-OLFM4, Cell Signaling Technology, Clone: D6Y5A, cat# 39141, 1:300
Mouse monoclonal anti-Vinculin, Sigma, clone: hVIN-1, cat# V9131, 1:1000
Goat anti-rabbit AF488, Invitrogen, Cat# A32731, 1:500
Donkey anti-rat AF647, Jackson ImmunoResearch, Cat# 712-605-153, 1:500
Rabbit polyclonal anti-ERK1/2, Cell Signaling Technology, cat# 9102S; 1:1000
Rabbit polyclonal Phospho-ERK1/2, Cell Signaling Technology, cat# 9101S; 1:1000
Biotinylated goat anti-mouse (Vector Labs, Cat #BA-9200, 1:300)
Biotinylated goat anti-rabbit (Vector Labs, Cat# is BA-1000, 1:300)
Goat anti-mouse IgG conjugated with Alexa Fluor 546 (Invitrogen, Cat# A-11003, 1:200)

Antibodies used in vivo:
InVivoMAb anti-mouse CD19, Bioxcell, clone: 1D3, cat# BE0150, injected i.p. at 300µg/mouse each time.
InVivoMAb rat IgG2a isotype control, anti-trinitrophenol, Bioxcell, clone: 2A3, catlog# BE0089, injected i.p. at 300 µg /mouse each time.
InVivoMAb anti-mouse CD8b, BioXcell, Clone: Lyt3.2, cat# BE0223, injected i.p. at 300 µg /mouse each time

Antibodies used in organoid culture:
Polyclonal Goat IgG Mouse Amphiregulin antibody, R&D systems, AF-989SP (1.5µg/ml)

Validation

For Rabbit anti-CYP27A1 antibody (obtained fromAbcam, clone: EPR7529, Cat# ab126785), we validated the antibody using our WT and Cyp27a1 KO mouse small intestine samples (dilution 1:200).

For all other antibodies listed above were validated by the manufacturer and/or by peer-reviewed article.

Antibody (Mouse anti-AREG) from Santacruz Biotechnology validation information could be found in the link: https://www.scbt.com/p/amphiregulin-antibody-g-4#citations

Mouse monoclonal anti-Vinculin from Sigma, the validation information and specification sheet can be found in this link: https://www.sigmaaldrich.com/SE/en/product/sigma/v9264

Donkey anti-rat AF647 from Jackson ImmunoResearch, the validation information and specification sheet can be found in this link: https://www.jacksonimmuno.com/catalog/products/712-605-153

Polyclonal Goat IgG Mouse Amphiregulin antibody from R&D system, the validation information and specification sheet can be found in this link: https://www.rndsystems.com/products/mouse-amphiregulin-antibody_af989

Biolegend antibodies validation information can be found in:
https://www.biolegend.com/en-us/quality/quality-control
For all flow cytometry antibodies, specificity testing of 1-3 target cell types with either single- or multi-color analysis (including positive and negative cell types). Once specificity is confirmed, each new lot must perform with similar intensity to the in-date reference lot. Brightness (MFI) is evaluated from both positive and negative populations. Each lot product is validated by QC testing with a series of titration dilutions. For all IHC antibodies, purified antibodies are tested for purity by SDS-PAGE gel electrophoresis. IgG antibodies are required to have purity >95%. Fluorophore and enzyme-conjugated antibodies follow strict manufacturing specifications to ensure performance. Each lot is validated by QC testing as stated on the TDS to confirm specificity and lot-to-lot consistency.

ThermoFisher (eBioscience) antibodies validation information can be found in:
https://www.thermofisher.com/se/en/home/life-science/antibodies/invitrogen-antibody-validation.html
Thermo Fisher Scientific's Invitrogen antibody validation process typically includes various methods to ensure antibody specificity and reliability, including:
Knockout Validation: Using knockout cell lines or tissues lacking the target protein to confirm antibody specificity by demonstrating the lack of signal in these samples; Transfection Validation: Demonstration of staining pattern changes when the target protein is transfected into cells that do not typically express the protein; Immunoprecipitation: Verifying antibody performance through immunoprecipitation techniques to confirm binding specificity; Peptide Array: Validating antibody binding specificity using peptide arrays containing the antigenic sequence; Overexpression Studies: Utilizing overexpression studies to verify antibody specificity and sensitivity; Western Blotting: Checking for specific band detection at the expected molecular weight. These validation methods help ensure that Thermo Fisher Scientific's antibodies provide accurate and reliable results for researchers.

BD biosciences antibodies validation information can be found in:
https://www.bdbiosciences.com/en-us/products/reagents/flow-cytometry-reagents/research-reagents/quality-and-reproducibility
The specificity is confirmed using multiple methodologies that may include a combination of flow cytometry, immunofluorescence, immunohistochemistry or western blot to test staining on a combination of primary cells, cell lines or transfectant models. All flow cytometry reagents are titrated on the relevant positive or negative cells. To save time and cell samples for researchers, test size reagents are bottled at an optimal concentration with the best signal-to-noise ratio on relevant models during the product development. To ensure consistent performance from lot-to-lot, each reagent is bottled to match the previous lot MFI. BD Biosciences ensures high-quality reagents through a meticulous quality control process, outlined as the following: ISO 9001 Compliance, R&D to Manufacturing Transfer, Standard Operating Procedures (SOPs) and Quality Control Testing. Newly manufactured lots undergo stringent quality control tests alongside accepted lots, ensuring that the performance of each new lot is reliable and consistent, providing researchers with assurance and confidence in their research outcomes.

Cell signaling antibodies validation information can be found in:
IHC antibodies: https://www.cellsignal.com/about-us/our-approach-process/antibody-validation-immunohistochemistry
Cell Signaling Technology (CST) offers over 800 antibodies validated for IHC, as well as IHC diluents, detection reagents, substrates and controls, to ensure that your IHC studies yield accurate and reproducible results. The determination of target specificity in immunohistochemical analysis requires multiple validation steps. CST scientists use a variety of methods, as appropriate, to validate each IHC-recommended antibody, ensuring that the staining you observe with each CST antibody is specific and believable.

Western blot antibodies:
https://www.cellsignal.com/about-us/our-approach-process/antibody-validation-western-blotting
The accuracy of western blot results relies heavily of the quality of the primary antibody employed in the immunoblotting. Cell Signaling Technology (CST) provides the highest quality primary and secondary antibodies available for western blotting. CST antibodies are produced in-house and validated extensively according to a rigorous protocol.

Abcam antibodies validation information can be found in:
https://go.myabcam.com/BiophysicalQuality
Abcam company uses a toolkit of tests established by the biopharma industry for therapeutic antibodies to assess sequence identity, sequence integrity, aggregation, purity, and concentration. These include liquid chromatography-mass spectrometry (LC-MS), dynamic light scattering (DLS) and high-performance liquid chromatography (HPLC). No other reagent supplier carries out this level of analysis, providing the highest level of assurance to researchers. The Biophysical QC includes: Recombinant technology – for exceptional batch-to-batch consistency, sensitivity and specificity; Extensive application testing – standard for all our antibodies, including immunohistochemistry (IHC) on tissue microarrays; Advanced validation – antibodies validated in techniques specifically of interest for their target, including mass cytometry, ChIC/CUT&RUN and biological activity, identified by our 'Advanced Validation' tag; Knock-out validation – performed using an extensive library of human knock-out cell lines.

R&D systems antibodies validation:
https://www.rndsystems.com/products/rd-systems-approach-antibody-quality
R&D Systems carefully tests every antibody we produce to ensure antibodies performance. Each antibody is manufactured under controlled conditions, undergoing rigorous quality control testing to ensure lot-to-lot consistency and outstanding performance in all applications listed on R&D systems datasheets. All antibodies are tested for cross-reactivity with closely related molecules using a variety of applications, including direct ELISA, to ensure specificity.

Animals and other organisms

Policy information about studies involving animals; ARRIVE guidelines recommended for reporting animal research

Laboratory animals	Mouse (Mus musculus)<br>All mice used were in C57BL/6J background. In all experiments animals used were between 8-20 weeks of age. Both males and female mice were used in the study, except for the AOM-DSS tumor experiments in Cyp27a1 KO mouse line. Specifically, C57BL/6J wilde type mice were purchased from TACONIC and maintained in specific pathogen free conditions at Karolinska Institutet (Sweden). Cyp27a1 line, Villin-Cre Yap1f/fTazf/f, Villin-Cre LXRαf/fβf/f line and Lgr5-eGFPIRES-creERT2 line mice were obtained as described in method section and maintained under specific pathogen free conditions at Karolinska Institutet (Sweden). Additionally, some experiments with Cyp27a1-/- and littermate controls where some mice were housed in an MPV-positive animal facility (the experiments with DSS administration and half of the irradiation experiments performed on Cyp27a1-/- and littermate controls. ApcMin/+ line mice were maintained and being used in experiments in Hamburg-Eppendorf University (Germany), Areg mice line were maintained and being used in experiments in Weizmann Institute of Science (Israel), all were bred and housed under specific pathogen free conditions.<br>Wild type or floxed control littermates were used as controls as indicated.
Wild animals	No wild animals were used in the study
Field-collected samples	Study did not involve field collected samples
Ethics oversight	Experiments using laboratory animals were approved and carried out in accordance to local the guidelines.<br>Experiments conducted at Karolinska Institutet (Sweden) were approved by Stockholm Regional Ethics Committee.<br><br>Experiments conducted in Hamburg-Eppendorf University (Germany) were approved by, the Institutional Review Board "Behörde für Justiz und Verbraucherschutz, Lebensmittelsicherheit und Veterinärwesen" (Hamburg, Germany).<br><br>Experiments conducted in Weizmann Institute of Science (Israel) were approved by the institutional guidelines for animal care and all experimental protocols were approved by the Institutional Animal Care and Use Committee (IACUC) of the Weizmann Institute.

Note that full information on the approval of the study protocol must also be provided in the manuscript.

Human research participants

Policy information about studies involving human research participants

Population characteristics	For human samples, samples were collected from healthy control (n= 28), ulcerative colitis (UC) active (n=39) and UC remission (n=27) from multiple sites such as terminal ileum, ascending colon and sigma/rectum from patients with IBD or suspicion of intestinal disease..<br><br>For human CRC samples, patients undergoing surgery for tumor resection had to be older than 18 years old and not have received chemotherapy or neoadjuvant therapy prior to total or partial colectomy. Tumor staging was classified according to the TNM classification (The Union for International Cancer Control; UICC).
Recruitment	For human samples, paired endoscopic biopsy specimens were obtained from the terminal ileum, ascending colon and sigma/rectum from patients with IBD or suspicion of intestinal disease.<br><br>For human CRC samples, samples from CRC patients from Colorectology Department, Clínica Las Condes, were included between 2015 and 2017. All patients signed informed consent forms approved by the institution (Cómite de ética de la investigación de CLC, O22019AA) and procedures were performed according to human experimental and clinical guidelines.<br><br>Potential biases include the recruitment source bias, where samples were gathered from specialized medical departments (from Germany and Chile), which may not represent the general population. Temporal bias could arise due to the specific period of sample collection from 2015 to 2017, impacting the applicability of results to other timeframes. Despite these biases, transparent reporting and comparative analyses are employed to mitigate their impact.
Ethics oversight	For human samples, the study was approved by the local ethical committee (EthikKommission der Ärztekammer Hamburg PV4444).<br><br>For human CRC samples, the study was approved by the institution (Cómite de ética de la investigación de CLC, O22019AA)

Note that full information on the approval of the study protocol must also be provided in the manuscript.

Flow Cytometry

Plots

Confirm that:

☒ The axis labels state the marker and fluorochrome used (e.g. CD4-FITC).

☒ The axis scales are clearly visible. Include numbers along axes only for bottom left plot of group (a 'group' is an analysis of identical markers).

☒ All plots are contour plots with outliers or pseudocolor plots.

☒ A numerical value for number of cells or percentage (with statistics) is provided.

Methodology

Sample preparation

For sorting of mouse primary intestinal epithelial cells, cells were first isolated by using EDTA treatment of minced tissues followed by mechanical dissociation and enzymatic digestion with TrpLE express to obtain single cell suspensions.

For sorting cells from organoids (for 10X scRNAseq), SI organoids on day5 were removed from the matrigel, cleaned with cold PBS and then digested with TrpLE Express to obtain the single cell suspensions.

For sorting intestinal epithelial, immune and double negative cells from mouse SI from steady state and irradiated mice, dissected small intestines were flushed with cold PBS and cut in to ~ 1cm pieces. To isolate the epithelial cells (IECs) and the intraepithelial lymphocytes (IELs), intestinal pieces were incubated in HBSS supplemented with 5% FCS, 15mM HEPES, 5mM EDTA and 1mM DTT for 30 minutes at 37°C under agitation at 800 rpm. The supernatant (called IEL fraction) containing IECs and IELs were filtered through 100μm cell strainers, centrifuged at 500x g for 5 minutes at 4°C and was enriched using 44%/67% Percoll gradient and washed for FACS analysis. To further isolate the cells from the lamina propria, the intestinal pieces were washed with PBS supplemented with 5% FCS and 1mM EDTA for 15 minutes at 37°C under agitation at 800 rpm. Next, the tissue pieces were digested in HBSS supplemented with 0.15mg/ml Liberase TL (Roche) and 0.1mg/ml DNaseI (Roche) at 37°C for 45 minutes under agitation at 800 rpm. The digested tissues were filtered through 100μm cell strainers and were washed for FACS analysis (called LP fraction).

Instrument

Cells were sorted either using FACS ARIA II (BD) or SONY SH800S cell sorter.

Software

FACS Diva and SH800S software. Cells were analyzed using FlowJo v10

Cell population abundance

The purity of sorted populations was dertermined by flow cytometry anaysis of sorted cells and frequencies for the gated population were above 90%. Sorted of intestinal epithelial cells from organoid culture was ~95-99% pure as it was obtained after 5 days of organoid culture. Purity of ex-vivo sorted intestinal epithelial cells, immune cells and double negative cells were ~90-95% pure based on the purity obtained from the FACS machine.

Gating strategy

Cells were gated using FSC-A, SSC-A followed by FSC-A and FSC-H to gate on the single cells. Gating strategy are provided in respective figures (Extended Data Figs. 2a, 2c, 9f) and in Supplementary Figs. 1 and 2.

☒ Tick this box to confirm that a figure exemplifying the gating strategy is provided in the Supplementary Information.
